# The efficacy and safety of Hanslim for obese patients: Study protocol for a multicenter, randomized, double-blind, multi-dose, placebo-controlled, phase IIb clinical trial

**DOI:** 10.1097/MD.0000000000012440

**Published:** 2018-09-21

**Authors:** Seunghoon Lee, Hyeonhoon Lee, Yeeun Cho, Jihye Kim, Jung Won Kang, Byung-Kwan Seo, Yong-Hyeon Baek, Jae-Dong Lee

**Affiliations:** a Department of Acupuncture and Moxibustion Medicine, Kyung Hee University Korean Medicine Hospital; b Department of Clinical Korean Medicine, Graduate School; c Department of Acupuncture and Moxibustion, College of Korean Medicine; d Oriental Medicine Research Center for Bone and Joint Disease, East-West Bone and Joint Research Institute, Kyung Hee University, Seoul, South Korea.

**Keywords:** Hanslim, herbal medicine, obesity, randomized clinical trial, study protocol

## Abstract

**Background:**

This study aims to evaluate the efficacy, safety, and appropriate dose of Hanslim, a Korean traditional herbal medicine, for obese patients, when compared to a placebo.

**Methods/design:**

This study is a randomized, double-blinded, multicenter, multidose, placebo-controlled, phase IIb clinical trial. A total of 165 obese patients with a body mass index (BMI) of more than 30 kg/m^2^ or obese patients with a BMI of 27 to 29.9 kg/m^2^ and one or more risk factors such as hypertension, diabetes, or hyperlipidemia will be enrolled. Participants will be randomly assigned to 1 of 3 groups (high-dose, low-dose, or placebo) with a 1:1:1 allocation ratio and will have 4 scheduled visits during the 12-week treatment period. The participants will be administered 2 tablets of Hanslim or placebo, 2 times per day. The difference in the proportion of participants who lost weight by more than 5% from their baseline at 12 weeks compared to the placebo group will be examined as the primary efficacy outcome. Secondary efficacy outcomes include differences in body weight, BMI, body-fat percentage, fat mass, skeletal-muscle mass, edema index, waist circumference, hip circumference, waist-hip ratio, serum lipid, blood glucose, C-reactive protein, and total score of Korean version of obesity-related quality of life after 12 weeks of treatment. Adverse events, laboratory test results, vital sings, and electrocardiography will be recorded to evaluate safety.

**Discussion:**

This is the first prospective clinical trial to explore the efficacy and safety of Hanslim for obese patients. If the results provide the appropriate dosage of Hanslim, this study would contribute to the confirmatory evidence for the use of Hanslim as a treatment for obesity needed to conduct a large-scale, phase III clinical trial. The results will be published in a peer-reviewed journal.

Trial registration: Clinical Research Information Service, ID: KCT0002193. Registered on January 6, 2017. https://cris.nih.go.kr/cris/search/search_result_st01_en.jsp?seq=7468

## 1 Introduction

Obesity is a global epidemic, associated with several medical complications that can decrease an individual's quality of life and even life expectancy.^[1]^ In Korea, 26.9% to 32.9% of the population is obese, according to the Korea National Health and Nutrition Examination Surveys.^[2]^ According to the World Health Organization, in 2016 more than 1.9 billion adults aged 18 years and over were overweight, and of these over 650 million were obese with approximately 39% of adults considered overweight.^[3]^ Left untreated, obesity may lead to the development of various complications such as diabetes mellitus, hypertension, heart disease, dyslipidemia, cerebrovascular disease, or cancer. It is also associated with reproductive disease, psychosocial disease, and musculoskeletal disease.^[4]^ This suggests that obesity is not only a matter of appearance, but an illness which should be managed to promote health and prevent disease.^[5]^ Some antiobesity agents have been discovered; however, they are associated with severe psychiatric and/or cardiovascular adverse events, highlighting the need for the development of other alternative drugs.^[6]^

Hanslim is a Korean traditional herbal formula developed in 2009 at Kyung Hee University Korean Medicine Hospital (KHUKMH). More than 5 million packs have been prescribed to overweight and obese patients in KHUKMH. The herbal formula originates from Wolbigachul-tang, which was reportedly used for patients with edema in the classic traditional medical literature, “Keumgueyoryak.”^[7]^ The components of Hanslim include *Ephedra intermedia* Schrenk, Gypsum fibrosum*, Atractylodes lancea* DC, and *Thea sinensis*. *Ephedra*, in particular, is considered a potential therapeutic agent for weight loss.^[8,9]^ It has recently been suggested that its anti-obesity effect is due to the modulation of gut microbiota.^[10]^ In previous experimental studies, Hanslim inhibited adipogenesis in 3T3-L1 cells through the reduction of triglyceride contents and lipid accumulation,^[11]^ and demonstrated an anti-obesity effect in diet-induced obese (DIO) animal models with weight loss, prevention of abdominal obesity, and improvement of abnormal lipid metabolism (Jae-Dong Lee, KMD, unpublished data, September 2012). Furthermore, in a retrospective clinical study of 205 obese patients, 54.1% of the patients succeeded in achieving 5% weight-loss after 3 months, and they did not complain of any unexpected adverse symptoms.^[12]^ In another retrospective study, Hanslim showed a pain-relieving effect in musculoskeletal pain patients after weight-reduction.^[13]^

These findings imply that Hanslim may become a novel drug for the treatment of obesity. However, the effect of Hanslim for obese patients has not been established with prospective clinical trials. Also, the right dose of Hanslim must be determined to prescribe the new drug safely and effectively. Therefore, we aim to design a large-scale clinical trial to evaluate the efficacy and safety of Hanslim for the treatment of obese patients and to determine the appropriate dosage.

## 2 Methods and design

### 2.1 Objective

The primary aim of this study is to determine if Hanslim is superior to a placebo by examining the proportion of participants who lost weight by more than 5% from baseline to 12 weeks of treatment. Secondary aims of this study are as follows: to determine if body weight, body mass index (BMI), body-fat percentage, fat mass, skeletal-muscle mass, edema index, waist circumference, hip circumference, waist-hip ratio, serum lipid, blood glucose, c-reactive protein, and total score of the Korean version of obesity-related quality of life (KOQoL) will improve more significantly in the group receiving Hanslim than in the group receiving the placebo after 12 weeks of treatment; to verify Hanslim is a safe treatment using laboratory tests, vital signs, and electrocardiography (ECG), and the collection of adverse events; and to determine which dosage of Hanslim is most effective.

### 2.2 Design and setting

This study is a multicenter, randomized, double-blind, multidose, placebo-controlled, phase IIb clinical trial. Eligible participants who sign informed consent voluntarily will be assigned randomly to 1 of 3 groups: high-dose (HD), low-dose (LD), or placebo, with a 1:1:1 allocation ratio. Participants will be administered 2 tablets of Hanslim or placebo 2 times per day for 12 weeks.

#### 2.2.1 Recruitment strategy

The participants will be recruited from 2 sites: KHUKMH at Hoegidong, and the hospital at Gangdong in Seoul, Korea. A total of 165 obese patients will be recruited through public mediums such as broadcast media, newspapers, the hospital's Internet homepage, and advertisements in nearby welfare centers and subway stations. Screening will continue until 165 participants are enrolled. The estimated recruitment period is from September 2017 to November 2018.

#### 2.2.2 Study plan

**Figure 1:**
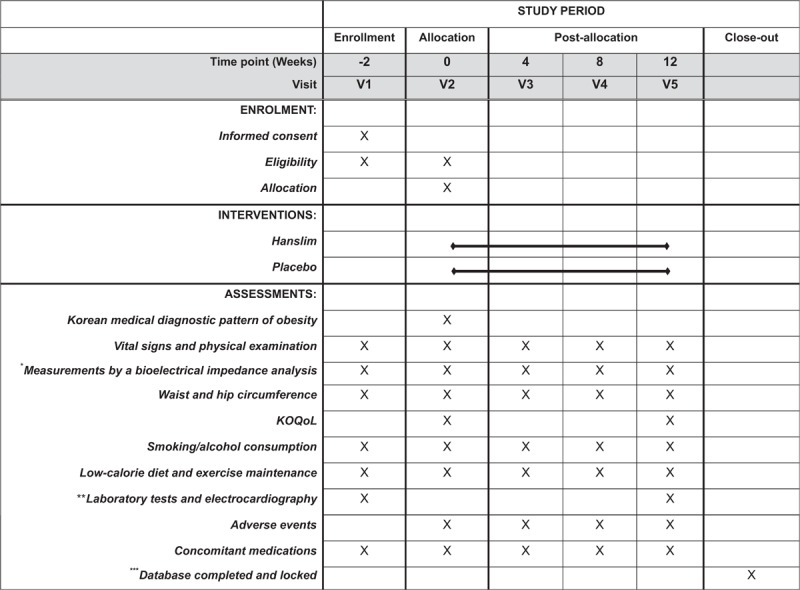
Schedule of enrolment, interventions, and assessments. KOQoL = Korean version of obesity-related quality of life, V = visit. ^∗^Body weight, height, body fat percentage, fat mass, skeletal muscle mass, and edema index will be measured. The height of participants will be measured at visit 1 only. ^∗∗^blood and urine test. ^∗∗∗^Documentation checked and finalized.

After participants consent voluntarily to the study, they will be screened at the first visit using several surveys, blood and urine tests, and ECG. The results of the tests will be conveyed by telephone, and participants who meet the inclusion criteria will be asked to attend a randomization visit within 2 weeks after the screening visit. If the participants are receiving treatment that may affect their weight or increase their blood pressure or heart rate, a 2-week wash-out period will be required. On the second visit, participants will be randomly assigned to one of the 3 groups and will be provided with the investigational drugs. Participants will take 2 tablets of Hanslim or placebo 2 times per day for 12 weeks. Additional visits will be made once per month. A total of 4 visits will take place during the treatment period. The efficacy and safety measurements will be taken 12 weeks after randomization. The detail time schedule is shown in Figure 1.

### 2.3 Types of participants

#### 2.3.1 Inclusion criteria

Participants who meet the following conditions will be included:

1. Males and females 19 to 65 years of age
2. Participants who meet one of the following criteria:
  a) Obese patients with a BMI of more than 30 kg/m^2^
  b) Obese patients with a BMI of 27 to 29.9 kg/m^2^ who have one or more risk factors such as hypertension, diabetes, or hyperlipidemia
3. Participants who agreed to follow a low-calorie diet during the clinical trial period
4. Participants who signed a consent form after being provided an explanation of the characteristics of this clinical trial

#### 2.3.2 Exclusion criteria

Participants will be excluded if any of the following conditions are satisfied:

1. Participants with endocrine diseases that may affect weight such as hypothyroidism or Cushing's syndrome.
2. Participants with heart diseases (heart failure, angina pectoris, or myocardial infarction), respiratory diseases (asthma or obstructive pulmonary disease), stroke, or ischemic heart failure.
3. Participants with malignant tumors within the last 5 years.
4. Participants who have narrow-angle glaucoma.
5. Participants who have cholelithiasis.
6. Participants who have resistant hypertension.
7. Participants who have severe renal function impairment (serum creatinine > 2.0 mg/dL).
8. Participants who have severe liver function impairment (alanine aminotransferase, aspartate aminotransferase, or alkaline phosphatase ≥ 2.5 times the upper limit of the normal range).
9. Participants who have uncontrolled diabetes with HbA1c more than 8%.
10. Participants with a neurologically or psychologically significant history of disease or who are currently suffering from the disease (schizophrenia, epilepsy, alcoholism, drug addiction, anorexia, hyperphagia, etc.).
11. Participants who have taken oral steroids with high doses (more than 30 mg prednisone per day or its equivalent).
12. Participants who have taken β-blocker or diuretic drugs for hypertension, anorexiants, laxatives, thyroid hormone, amphetamine, cyproheptadine, phenothiazine, contraceptive or female hormones within the last 3 months that may affect weight.
13. Participants who have taken other forbidden treatments (insulin, antidepressants, selective serotonin reuptake inhibitors (SSRIs), barbiturate, antipsychotics, or drugs concerned with abuse).
14. Participants who underwent anatomical changes, such as incision, which make it difficult to conduct physical measurements.
15. Participants who underwent surgeries for weight reduction (e.g., gastroplasty or gastrectomy)
16. Pregnant women, breastfeeding women, women planning a pregnancy and those who do not agree to use adequate contraception such as oral contraceptives, a hormone transplant, an intrauterine device, spermicide, condoms, or celibacy.
17. Participants who have lost more than 10% of their body weight within the last 6 months.
18. Cessation of smoking within the last 3 months or an irregular smoking habit.
19. Participants of other interventional clinical studies or participants who have taken other investigational drugs within the last 1 month.
20. Participants who are judged as unable to comply with the study according to the findings of the clinical investigator.

### 2.4 Randomization and allocation concealment

An independent, blinded statistician will generate random numbers using the randomization method of SAS Version 9.2 (SAS institute Inc., Cary, NC). One hundred sixty-five eligible participants will be assigned to each group in a 1:1:1 ratio. Allocation concealment will be maintained throughout the study, as participants will be given identically packaged, consecutively numbered drug containers.

### 2.5 Blinding

The participants, outcome assessors, study monitors, data managers, and statisticians will be blinded to the allocation. If possible, participants will be advised not to compare the investigational drugs with those of other participants. The blinding test will be conducted during the last visit. The blinding will be maintained until all 165 participants complete the study, the database is locked, and data analysis has been completed.

Separate emergency codes will be opened only in cases when researchers must inform the participants regarding the investigational drugs they received (e.g. to determine a subsequent treatment to manage serious adverse events [AEs]).

### 2.6 Intervention

The total dose of each tablet of Hanslim is 865 mg, of which 240 mg is dried herbal extract in the low-dose tablet, and 480 mg is dried herbal extract in the high-dose tablet. The herbal extract was obtained from *Ephedra intermedia* Schrenk, Gypsum fibrosum*, Atractylodes lancea* DC, and *Thea sinensis L.* The raw material and the sample were deposited in the herbarium of Quality Control of the Hanpoong Pharm and Foods Co., Ltd. (Wanju-Gun, South Korea). The raw materials were extracted with water at 90°C to 100°C for 3 hours and filtered. The process was repeated 2 times. A high-performance liquid chromatography analysis was performed to quantify the chemical constituents of the herbal extract. The herbal extract of Hanslim was controlled to contain more than 5.50 mg/g Ephedrine and pseudoephedrine (C_10_H_15_NO), 18.33 mg/g Caffeine (C_8_H_10_N_4_O_2_), and 49.70 mg/g epigallocatechin gallate (EGCG, C_22_H_18_O_11_). To test the purity, heavy-metal tests, residual-pesticide tests, and a microbial-limit test were conducted according to the Korea Pharmacopeia. The tests looked for: total heavy metals, lead (Pb), arsenic (As), dichlorodiphenyltrichloroethane, benzene hexachloride, aldrin, dieldrin, endrin, the total number of aerobic microorganisms, the total number of fungi, *Escherichia coli, Salmonella, Pseudomonas aeruginosa,* and *Staphylococcus aureus*.

The placebo tablets that will be used in the trial are comprised of cellulose, cornstarch, and diluting agents. They are colored with food coloring to match the Hanslim tablets. The Hanslim and placebo tablets are manufactured to have no difference in shape, color, size, odor, or taste. All manufacturing processes for the Hanslim and placebo tablets were conducted at Hanpoong Pharm and Foods Co., Ltd., according to the Korean Good Manufacturing Practices.

Participants will take 2 tablets of Hanslim (low or high dose) or placebo, 2 times per day for 12 weeks. This dosage regimen was determined after referring to preclinical studies. During the 12 weeks of treatment, participants will comply with the study protocol by visiting the clinic every 4 weeks and completing scheduled examinations. If AEs due to the investigational drugs occur, researchers will alter the dose or frequency according to the standard operating procedures (SOPs).

### 2.7 Permitted and prohibited concomitant treatments

Concomitant drugs that are unrelated to obesity and that have been administered for more than 30 days before the screening visit will be permitted with the treatment regimen. Prohibited drugs include medications or health foods for the purpose of weight-loss such as weight-loss agents and dietary supplements, agents that may affect obesity such as insulin, antidiabetics, high-dose oral steroids (≥30 mg), thyroid hormones, cyproheptadine, phenothiazine, diuretics, β-blocker as antihypertensive agent, contraceptives, female sex hormones, amphetamine, antidepressants, SSRIs, barbiturate, and antipsychotics, and 3) agents that can increase blood pressure or heart rate.

### 2.8 Outcomes

#### 2.8.1 Primary outcome measurement

The difference in the proportion of participants who lost weight by more than 5% from their baseline compared to the placebo group, following the Food and Drug Administration guidelines regarding main outcomes in clinical trials evaluating weight-loss drugs,^[14]^ at 12 weeks after randomization will be used as the primary outcome measurement.

#### 2.8.2 Secondary outcome measurements

Secondary outcomes include the difference in body weight at 4 and 8 weeks after randomization, BMI, body-fat percentage, fat mass, skeletal-muscle mass, edema index, waist circumference, hip circumference, and waist-hip ratio at 4, 8, and 12 weeks after randomization, serum lipid, blood glucose, C-reactive protein, and total score of KOQoL at 12 weeks after randomization. Body weight, height, body-fat percentage, fat mass, skeletal-muscle mass, and edema index will be measured using a bioelectrical impedance analysis (InBody 720, InBody Co., Seoul, South Korea). Waist circumference will be measured horizontally, at the middle of the lowest rib and the iliac crest. Hip circumference will also be measured horizontally, at the largest part of the hip.

#### 2.8.3 Safety outcome measurements

Safety will be examined through vital signs, physical examinations, laboratory (blood and urine) tests, ECG, and adverse events. Blood tests will include hemoglobin, hematocrit, red blood cell count (RBC), white blood cell count (WBC), platelet count, total protein, albumin, total bilirubin, liver function tests, uric acid, blood urea nitrogen (BUN), creatinine, BUN/creatinine ratio, fasting plasma glucose, creatinine kinase, hemoglobin A1c, thyroid-stimulating hormone, free thyroxine, hepatitis B virus surface (HBs) antigen, anti-HBs antibody, triglyceride, total cholesterol, low-density lipoprotein cholesterol, high-density lipoprotein cholesterol, and C-reactive protein. Urine tests will include specific gravity, pH, protein, glucose, ketone, urobilinogen, bilirubin, nitrite, RBC, and WBC.

### 2.9 Sample size

This study was developed to test 2 hypotheses as follows:

H_01_: *p*_1_ = *p*_2_
H_11_: *p*_1_ ≠ *p*_2_
H_02_: *p*_1_ = *p*_3_
H_12_: *p*_1_ ≠ *p*_3_
*p*_1_ = proportion of participants in the placebo group who lost weight by more than 5% after 12 weeks of treatment
*p*_2_ = proportion of participants in the LD Hanslim group who lost weight by more than 5% after 12 weeks of treatment
*p*_3_ = proportion of participants in the HD Hanslim group who lost weight by more than 5% after 12 weeks of treatment

Based on previous studies, we assumed the proportion of the placebo group will be 15%.^[15,16]^ In a retrospective chart review by Jo et al^[12]^ that investigated the weight-loss effect of Hanslim on obese patients (n = 205) at KHUKMH, the proportion of participants who lost weight by more than 5% after 12 weeks of treatment was 53.7% (95% Confidence Interval, 46.83% to 60.48%). Considering that previous herbal studies for weight loss showed a 10–20% lower effect than the estimated effect of the herbal formula before the study,^[15,17]^ we assumed that the proportion of participants in the Hanslim group who lost weight by more than 5% may be 43%, approximately 10% lower than 46.83%, which is the lower limit of the confidence interval in the result of the retrospective study. The allocation ratio is 1:1:1. After taking into account that the estimated effect rate of the treatment group and the control group is 43% and 15%, respectively, 41 participants are required for each group with a 2-sided significance level of 5% (α = 0.05) and 80% power (1−β = 0.8). Consequently, a sample size of 55 per group and a total number of 165 will be included, accounting for a possible 25% drop-out rate.

### 2.10 Data management

Researchers who have read and understood the SOPs will obtain written consent, as well as collect and manage data from the participants. Participants will be encouraged to complete the study. If a participant must withdraw, the test for the last visit will be conducted with the participant's agreement. If a follow-up investigation is required, researchers will contact the participant by telephone as necessary. After the end of the trial, data entry will be proceeded by a double entry, and the matching work will be conducted after reviewing inconsistent data. When the data are matched, a data clarification form (DCF) will be completed and validated, the resolution will be reflected in the data, and medical coding will be performed through MedDRA (ver 21.0).

### 2.11 Statistical analysis

The analysis set will consist of a full analysis set (FAS), a per protocol (PP) set, and a safety set. The FAS set will include all randomized participants who received any study treatment and had at least one assessment after treatment. The PP set will include only participants who completed the treatment according to the protocol. The minimum compliance rate of participants receiving the investigational drugs for the PP set is 80%. The safety set will include any participants who were randomly assigned and received at least one investigational drug. FAS analysis will be the main analysis; it will be compared to the PP analysis as a sensitivity analysis. An interim analysis will not be performed.

For the descriptive analysis, Student's *t*-test or a Wilcoxon rank sum test will be performed for continuous data, and a chi-squared test or Fisher's exact test will be performed for categorical data.

For a confirmatory analysis, after calculating the proportion of participants who lost more than 5% from the baseline after 12 weeks of treatment, the rate differences between the 2 groups (LD group vs placebo group and HD group vs. placebo group), the primary outcome measurement, will be compared using the Chi-square test or Fisher's exact test. In the secondary outcome measurements, category data will be analyzed following the same methodology as that used in the primary outcome measurement, and continuous data will be analyzed using Student's *t*-test or the Wilcoxon rank-sum test. Where baseline measurements or study site statistically differ among groups, analysis of covariance with the baseline measurements as a covariate for continuous data or the Cochran–Mantel–Haenszel test with study site as strata for categorical data will be conducted. Additionally, repeated measure analysis of variance will be used to identify any trend changes.

A safety assessment will be performed for all AEs that occur during the study period. The incidence of AEs, AEs leading to withdrawal, and serious AEs will be summarized by group and analyzed using Fisher's exact test or the Chi-square test.

All statistical analyses will be conducted by a statistician blinded to group allocation with the SAS package (Version 9.2, SAS Institute Inc., Cary, NC). The level of significance will be set at 5%, and a 2-tailed test will be used. The last-observation-carried-forward method will be used to replace the missing values in the FAS set.

### 2.12 Data monitoring and audit

The contract research organization, MediHelpLine Co., Ltd (Seoul, South Korea) will conduct regular monitoring to ensure quality control of the data. The clinical research associate will monitor the written informed consent documents, protocol compliance, data quality, and overall trial progress during the study period. The study will proceed until 165 participants are randomly assigned and have completed the study. In the determination of human research protection program at KHUKMH, internal audit may be conducted before completion of the study.

### 2.13 Adverse events

Researchers will record symptoms that the participants report during the treatment period as adverse events on a case report form at every visit. A causal relationship between the investigational drugs and AEs will be assessed using a 6-grade scale (1 = definitely related, 2 = probably related, 3 = possibly related, 4 = probably not related, 5 = definitely not related, and 6 = unknown), and the severity of the AEs will be scored using a 4-Likert scale (mild, moderate, severe, or life threatening). The expected AEs involve the dermis (redness and rash), autonomic nervous system (insomnia and dizziness), gastrointestinal system (nausea, vomiting, constipation, diarrhea, and dyspepsia), circulatory system (palpitation and tachycardia), urinary system (dysuria), and others (headache, dry mouth, and hand tremor).^[12,18]^ If AEs due to the investigational drugs occur, researchers will may alter the dose or frequency according to the SOP.

### 2.14 Ethics approval and consent to participate

The protocol and relevant documents of this study were reviewed and approved by the Ministry of Food and Drug Safety (MFDS) and the institutional review boards (IRB) of KHUKMH at Hoegidong (KOMCIRB-161014-BR-056) and the hospital at Gangdong (KHNMCOH 2016–10-014). This study was planned according to tenets of the Declaration of Helsinki and the Korean Good Clinical Practice Guidelines.

If major changes are necessary, they will be reported to the MFDS and the IRB for approval. We will include only applicants who voluntarily agree by written informed consent after a full explanation of the study prior to screening. The trial is registered with the Clinical Research Information Service (KCT0002193). Data related to this clinical trial will be kept confidential, and participant information will be processed anonymously.

## 3 Discussion

This is the study protocol of a multicenter, randomized, double-blind, multi-dose, placebo-controlled, parallel, phase IIb clinical trial for Hanslim use with obese patients. This study aims to evaluate the efficacy, safety, and appropriate dose of Hanslim compared to placebo after 12 weeks of treatment.

The recent clinical practice guideline in Korea recommended that Taeeumjowui-tang or Bangpungtongseong-san be considered in the treatment of adult obese patients with moderate-certainty because the herbal formulas showed significant effects to reduce body weight and waist circumference.^[19]^ However, these are composed of a number of herbs, which makes it difficult to standardize the use of herbal medicine. In addition, since *Ephedra* has been reported to have considerable side effects,^[12,18]^ it is necessary to decrease the dosage while maintaining its effect for weight loss. Therefore, on the basis of previous experimental studies and clinical experience, we developed Hanslim to maximize the efficacy of *Ephedra* as well as decrease the expected side effects by reorganizing the proportion of herb components in the original herbal formula, Wolbigachul-tang.^[7]^ In some previous studies,^[12,13]^ it was found that Hanslim had anti-obesity effect; compared to the active control, it reduced body weight and fat as well as improved the total cholesterol, high-density lipoprotein cholesterol, low-density lipoprotein cholesterol, and blood glucose level.

To our knowledge, this is the first prospective clinical trial to explore the efficacy and safety of Hanslim for obese patients. If the results provide the appropriate dosage of Hanslim, this study would contribute to the confirmatory evidence for Hanslim as a treatment of obesity needed to conducting a large-scale, phase III clinical trial.

## Acknowledgments

We would like to thank all of the members of the research team (Korean medicine doctors, pharmacists, and clinical research coordinators) at KHUKMH at Hoegidong and Gangdong.

## Author contributions

**Conceptualization:** Seunghoon Lee, Jae-Dong Lee

**Funding acquisition:** Jae-Dong Lee

**Methodology and project administration:** Seunghoon Lee, Jae-Dong Lee

**Writing – original draft:** Seunghoon Lee, Hyeonhoon Lee, Jihye Kim, Yeeun Cho

**Writing – review & editing:** Jung Won Kang, Byung-Kwan Seo, Yong-Hyeon Baek
